# Application of microalgae *Chlamydomonas applanata* M9V and *Chlorella vulgaris* S3 for wheat growth promotion and as urea alternatives

**DOI:** 10.3389/fmicb.2022.1035791

**Published:** 2022-11-29

**Authors:** Mekiso Yohannes Sido, Yinping Tian, Xiaogai Wang, Xinzhen Wang

**Affiliations:** ^1^Key Laboratory of Agricultural Water Resources, Hebei Key Laboratory of Soil Ecology, Center for Agricultural Resources Research, Institute of Genetics and Developmental Biology, Chinese Academy of Sciences, Shijiazhuang, China; ^2^College of Agriculture, Wachemo University, Hosaena, Ethiopia; ^3^School of Life Science and Engineering, Handan University, Handan, China

**Keywords:** chemical fertilizer, microalgal fertilizer, *Chlamydomonas applanata* M9V, *Chlorella vulgaris* S3, crop growth promotion, urea alternative

## Abstract

Excessive use of chemical fertilizers to meet the global food demand has caused extensive environmental pollution. Microalgae can be used to enhance agricultural crop production as a potentially sustainable and eco-friendly alternative. In this study, *Chlamydomonas applanata* M9V and *Chlorella vulgaris* S3 were isolated from the soil and mass-cultured for use as microalgal fertilizers. The influence of microalgae M9V and S3 on the growth of wheat (*Triticum aestivum* L.) and soil properties was evaluated and compared with that of chemical urea fertilizer. A pot experiment was conducted with six treatments, i.e., living M9V (M9VL), dead M9V (M9VD), living S3 (S3L), dead S3 (S3D), urea fertilizer (urea), and control without fertilizer (control). M9VL was found to have the best effect on wheat growth promotion, followed by M9VD and S3D. In addition, M9VL resulted in the highest enhancement of shoot fresh weight (166.67 and 125.68%), root dry weight (188.89 and 77.35%), leaf length (26.88 and 14.56%), root length (46.04 and 43.93%), chlorophyll *a* (257.81 and 82.23%), and chlorophyll *b* contents (269.00 and 247.27%) comparing to the control and urea treatments, respectively. Moreover, all microalgal fertilizer treatments increased soil organic matter (SOM) by 1.77–23.10%, total carbon (TC) by 7.14–14.46%, and C:N ratio by 2.99–11.73% compared to the control and urea treatments. Overall, this study provided two microalgae strains, M9V and S3, that could promote wheat growth and improve soil properties, thus highlighting the use of microalgae as biofertilizers to reduce the use of chemical fertilizers and promoting sustainable agricultural production.

## Introduction

The global food demand has been increasing rapidly, with a predicted increase of 60–110% in the global crop demand by 2050, so huge are the environmental impacts expected from the increased agricultural production to meet this demand correspondingly (Bruinsma, 2009; Tilman et al., 2011; Odegard and van der Voet, 2014; Rockström et al., 2017; Nascimento et al., 2019). Chemical fertilizers have been used on crops to increase food production for a long time; this increases the input cost of farming, reduces the utilization rate of soil fertilizers, and causes soil agglomeration and hardening, biodiversity loss, and lower productivity. This will seriously affect the sustainable and stable development of agricultural production (Garcia-Gonzalez and Sommerfeld, 2016; Rahman and Zhang, 2018). In addition, the large amount of fertilizer accumulated in the soil causes large-scale soil and water pollution through surface runoff and leaching, which seriously endanger the natural environment and human health. Thus, in the coming decades, one fundamental challenge will be preventing food shortages without accelerating environmental pollution and ecological degradation (Godfray et al., 2010; Odegard and van der Voet, 2014; Garcia-Gonzalez and Sommerfeld, 2016).

Microalgae, autotrophic plants with fast photosynthesis, fast reproduction, and strong environmental adaptability, whose cell metabolism results in the production of fat, protein, pigment, and polysaccharides, are considered to have great potential for solving major practical challenges, such as the lack of healthy food, increasing greenhouse effect, pollution of the ecological environment, and energy crisis (Michalak et al., 2017; Chiaiese et al., 2018; Behera et al., 2021). They are widely used in fuel, food, medicine, cosmetics, animal feed, and sewage purification (Zhu et al., 2013; Yaakob et al., 2014; Michalak et al., 2017; Moreno-Garcia et al., 2017; Morais et al., 2021; Moreira et al., 2022), but their use is rarely recognized in agricultural production (Garcia-Gonzalez and Sommerfeld, 2016). Moreover, these studies on microalgal resources mainly focused on water environments, such as seawater and fresh water, while few have focused on more complex and diverse soil environments that contain rich biological resources. Many microalgal resources derived from the soil environment, especially those beneficial to agricultural production, are yet to be developed.

Some microalgae have been proven to have a robust effect on the root system of crops and enhance crop yield and quality by improving soil structure or fertility, promoting the activity of beneficial soil microorganisms, and balancing the soil micro-ecosystem (Ronga et al., 2019; Martini et al., 2021). In this study, we aimed to isolate and identify microalgal strains from soil environments and assess their prospects in agricultural crop production by analyzing the influence of microalgae on wheat growth and soil properties. This study will support the use of microalgae as biological fertilizers that have great potential to meet various needs, such as reducing the application of chemical fertilizers, increasing food production, and maintaining environmental and ecological health in agricultural production.

## Materials and methods

### Microalgae isolation and purification

**FIGURE 1:**
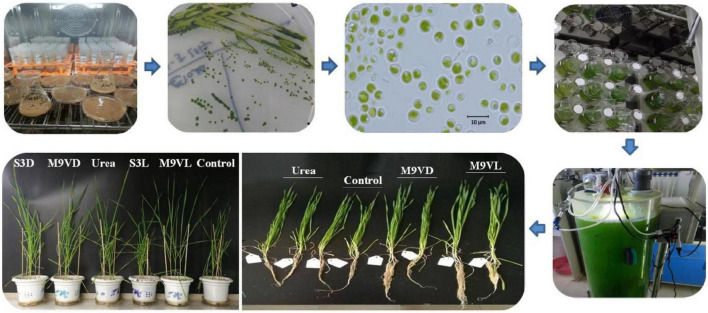
Isolation, purification, cultivation, and the application to pot experiment of microalgae *Chlamydomonas applanata* M9V and *Chlorella vulgaris* S3.

Isolation and purification of the microalgal strains were conducted using saline (38°10′02″N, 117°33′49″E) and grassland soils (37°52′44″N, 114°15′49″E) in Hebei Province, China. Soil samples were taken from 0 to 10 cm of the topsoil, carefully transported to the laboratory, and stored at 4°C. To isolate the microalgae from the soil, 1 g fresh soil was mixed thoroughly with 10 mL sterilized distilled water by vortexing for 30 min. The mixture was supplied with the Allen Arnon medium (AA medium) for enriching the microalgae and incubated for a week at 25.5°C after shaking at 200 rpm for 24 h (Figure 1; Allen and Arnon, 1955). The supernatant was carefully transferred into sterilized flasks containing AA medium with imipenem at a final concentration of 100 μg mL^–1^, which could inhibit prokaryotic cell growth. The culture was shaken at 150 rpm for 3 weeks at a light intensity of 100 μmol photons m^–2^ s^–1^ for 12 h per day at 25°C. Then, 1 mL of the mixed culture was serially diluted. Hundred microliters of each serial dilution was coated on 1.5% agar solid AA medium with imipenem for microalgae growth with a light intensity of 100 μmol photons m^–2^ s^–1^ at 25°C until green colonies were observed. Single colonies were repeatedly selected and streaked on a solid AA medium and incubated until the single colonies consistently had the same appearance and morphology as the microalgal cells under the microscope (BX3-URA, Olympus, Japan) (Figure 1).

The AA medium was prepared from a mixture of +pi and −pi stock solutions as follows. The +pi stock solution was prepared from autoclaved anhydrous K_2_HPO_4_ (42.8 g L^–1^), and the −pi stock solution was prepared from four stock solutions that were mixed at a ratio of 1:1:1:1, including autoclaved macroelement stocks of 40 g L^–1^ MgSO_4_⋅7H_2_O, 12 g L^–1^ CaCl_2_⋅H_2_O, 40 g L^–1^ NaCl, and filter sterilized microelement stock (1,090 mL double-distilled water, 160 mL Fe-EDTA, 360 mg MnCl_2_⋅4H_2_O, 36 mg MoO_3_ 85% purity, 44 mg ZnSO_4_⋅7H_2_O, 15.8 mg CuSO_4_⋅5H_2_O, 572 mg H_3_BO_3_, 4.6 mg NH_4_VO_3_ (NH_4_^+^ metavanadate), and 8 mg CoCl_2_⋅6H_2_O). For microalgal growth, the +pi solution was used as 6.25 mL L^–1^ for solid plate culture and 3.1 mL L^–1^ for liquid culture, and the −pi solution was used as 25 mL L^–1^ for solid plate and 6.30 mL L^–1^ for liquid culture (Allen and Arnon, 1955).

### DNA extraction, PCR amplification, sequencing, and phylogenetic analysis

Genomic DNA was extracted from a 2 mL microalgal culture using an E.Z.N.A. @ HP fungal DNA kit (D3195-01, Omega, United States). The quantity and quality of the extracted DNA were tested using a NanoDrop One instrument and 1% (w/v) agarose gel electrophoresis. The small subunit ribosomal DNA (SSU rDNA) sequence was amplified using primer set 18F (5′-TGGTTGATCCTGCCAGT-3′) and 18R (5′-TGATCCTTCTGCAGGTTCACC-3′) (Song et al., 2016). The 50-μL PCR mixture contained 25 μL of Premix Ex Taq, 0.5 μL of each primer (10 μM), 3 μL of template DNA, and 21 μL of ddH_2_O. The PCR was conducted with an initial denaturation at 94°C for 5 min, followed by 32 cycles of 50 s at 94°C, 50 s at 55°C, and 90 s at 72°C (Song et al., 2016). The PCR amplification products were checked by 1% (w/v) agarose gel electrophoresis at 180 V for 15 min with a 2,000 bp DNA ladder. Finally, the amplified PCR products were sequenced by Sangon Biotechnology Inc. (Shanghai, China). The closest relatives of SSU rDNA sequences of microalgal strains M9V and S3 were examined using the BLASTn search program on the National Center for Biotechnology Information (NCBI) website.^1^ Reference sequences from microalgal organisms were retrieved from GenBank. After the sequences were aligned with CLUSTALX 1.81 (Thompson et al., 1997), neighbor-joining phylogenetic trees were constructed using the software MEGA7 with 1,000 bootstrap replicates (Kumar et al., 2016).

### Pot experiment

The experiment was conducted in pots containing 3.0 kg fresh and 2-mm-diameter mesh-sieved soil. Four healthy wheat plants of a similar size of variety Kenong199 (KN199, provided by the State Key Laboratory of Plant Cell and Chromosome Engineering) were planted in each pot and cultured in a controlled environment for 56 days with a relative humidity of 65 ± 5% and light intensity of 248 μmol photons m^–2^s^–1^ for a 16 h photoperiod per day at 25°C (Figure 1). In this pot experiment, the growth period of wheat plants growing for 56 days could be judged as seedling stage according to their characteristics of no-tillers and less than nine leaves. The main reason for this phenomenon was that the objective of our experiment was to investigate the relative difference in wheat growth between the treatment with microalgal fertilizer and the control and urea treatments without microalgal fertilizer, so the vernalization treatment of wheat seedlings was not carried out, and the growth environment was kept at 25°C without temperature difference. Six treatments (i.e., M9VL, M9VD, S3L, S3D, urea, and the control), were laid out in a completely randomized design (Figure 1). All treatments were repeated two times. To obtain a large amount of microalgae as biofertilizer, we used a 20 L volume light bioreactor to cultivate the microalgae *Chlamydomonas applanata* M9V and *Chlorella vulgaris* S3 with a light intensity of 100 μmol photons m^–2^s^–1^ for a 12 h photoperiod per day at 25°C. M9VD and S3D were obtained from equal quantities of M9VL and S3L processed in an autoclave sterilizer at 121°C for 30 min. Urea treatment was employed by using 0.39 g urea fertilizer at 0.18 g N per pot (the recommended rate of N fertilizer, 120 kg N ha^–1^, roughly calculated according to 2.0 × 10^6^ kg soil ha^–1^) (Oad and Buriro, 2004; Maurya et al., 2016). Based on equal N nutrient content with urea treatment at 0.18 g N per pot, 3.28 g dry weight of microalgae M9V biomass was applied to each pot for the M9VL and M9VD treatments, and 3.75 g dry weight of microalgae S3 biomass was applied to each pot for the S3L and S3D treatments. All the treatments, including the control, were supplemented with the AA medium to normalize the effect of the AA medium on wheat growth. The recommended rate of phosphorus (60 kg P_2_O_5_ ha^–1^) was supplied with triple super phosphate to all treatments (Maurya et al., 2016; Xiao et al., 2021).

### Soil property analysis

The measurement of soil chemical characteristics, including pH, total carbon (TC), total nitrogen (TN), and soil organic matter (SOM) contents, was conducted according to the methods previously described by Lu (1999) and Chu and Grogan (2010). To determine the soil pH value, a soil to CO_2_-free water ratio of 1:2.5 was used and the pH was measured using a pH meter (PHS-3C, Shanghai INESA, China). The soil samples were air-dried and sieved with a 2 mm mesh, and then ground using a mortar and pestle to determine soil TC, TN, and SOM contents. The TC and TN contents of the soil were measured using an elemental analyzer (Vario Pyro, Elementar, Germany). The SOM content was measured using the K_2_Cr_2_O_7_ oxidation method.

### Data collection on wheat growth parameters

After the potting soil was saturated with tap water to loosen the root attachment to the soil, we washed the wheat roots gently and thoroughly with tap water to keep them intact. The four wheat plants in each pot were separated into independent individuals, and finally, we obtained eight wheat plants for data collection for each treatment. Data on shoot fresh weight, leaf length, and root length of wheat plants were collected first, and data on root dry weight were collected after the wheat plant was dried in an oven. The leaf length was recorded from the longest leaf of each wheat plant. To determine the chlorophyll *a*/*b* and carotenoid contents, 0.5 g fresh leaf samples were homogenized using a mortar and pestle with 10 mL 80% acetone, and the extract was centrifuged at 650 × *g* for 5 min at 4°C. Then, 1 mL supernatant was gently transferred into a new tube and diluted by adding 9 mL 80% acetone. The absorbance of the diluted extract was measured at 663.2 nm, 646.8 nm, and 470 nm, and then chlorophyll *a*/*b* and carotenoid contents were calculated using the method described by Wellburn (1994).

### Microalgal element composition analysis

To remove medium nutrients, the biomass of the microalgae *C. applanata* M9V and *C. vulgaris* S3 was washed with sterilized double-distilled water and centrifuged at 2,600 × *g* for 10 min. Then, the biomass was dried at 65°C and ground, and elemental composition, i.e., N, C, P, K, Fe, Zn, and Mn, was evaluated using an atomic absorption spectrophotometer (AAS, ZEEnit 700P, Analytik Jena, Germany) and elemental analyzer (Vario Pyro, Elementar, Germany).

### Statistical analysis

The data are described based on the mean with standard deviation. Statistical analysis was performed using one-way analysis of variance (ANOVA) with the Statistical Analysis Software (SAS) version 9.2, and the significance level was defined as *p* < 0.05 level and labeled with letters based on Tukey’s test. Origin 2021 software was used in plotting.

## Results

### Phylogeny of microalgal strains M9V and S3

**FIGURE 2:**
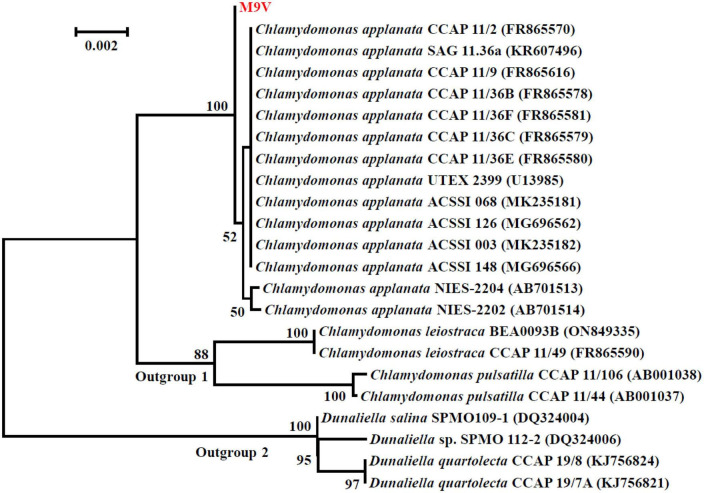
Neighbor-joining phylogenetic tree of the small subunit ribosomal DNA (SSU rDNA) sequence of microalgae M9V and the reference sequences retrieved from GenBank. Numbers in parentheses are the accession numbers of reference sequences in the National Center for Biotechnology Information (NCBI) database.

**FIGURE 3:**
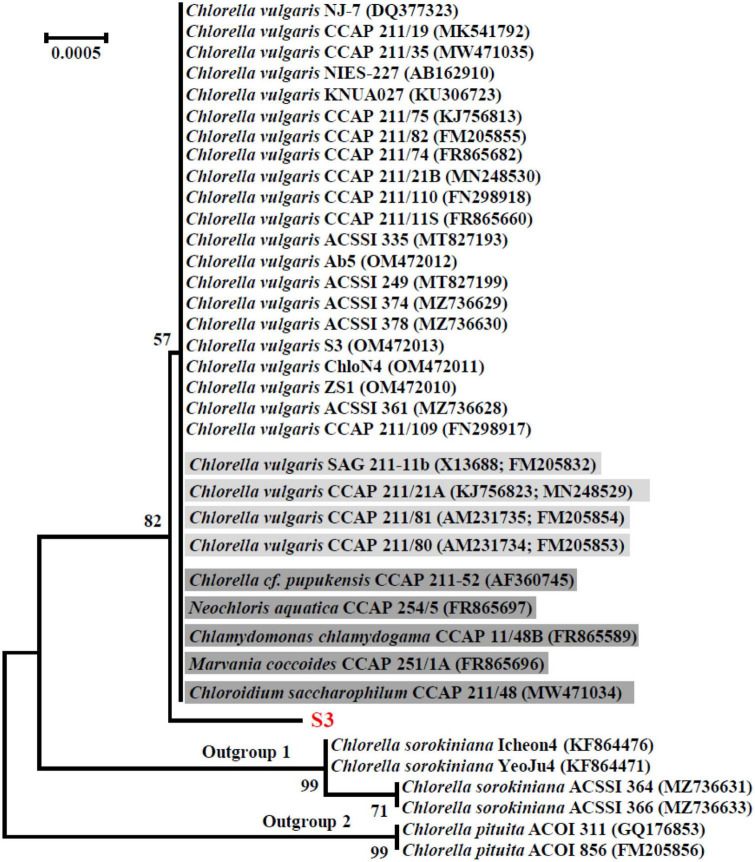
Neighbor-joining phylogenetic tree of the small subunit ribosomal DNA (SSU rDNA) sequence of microalgae S3 and the reference sequences retrieved from GenBank. Numbers in parentheses are the accession numbers of reference sequences in the NCBI database.

The SSU rDNA sequences of microalgal strains M9V and S3 were deposited into the NCBI database with accession numbers MK793578 and MK652782, respectively. In the NCBI database, the SSU rDNA sequence of microalgae M9V has the highest 99.94% identity with 12 reference microalgal sequences, and the SSU rDNA sequence of microalgae S3 has the highest 99.88% identity with 34 reference microalgal sequences. These reference sequences are described as SSU rDNA, 18S rDNA, and genomic DNA containing 18S rRNA gene, ITS1, 5.8S rRNA gene, ITS2, 28S rRNA gene in the NCBI database. The SSU rDNA sequence of microalgae M9V was clustered with those 12 reference sequences from *C. applanata* organisms CCAP 11/2, SAG 11.36a, CCAP 11/9, CCAP 11/36B, CCAP 11/36F, CCAP 11/36C, CCAP 11/36E, UTEX 2399, ACSSI 068, ACSSI 126, ACSSI 003, and ACSSI 148, as well as two reference sequences from *C. applanata* organisms NIES-2204 and NIES-2202 of lower identity with 100% bootstrap support value in the phylogenetic tree (Figure 2). Thus, the microalgal strain M9V was named *C. applanata* M9V. The SSU rDNA sequence of microalgae S3 was clustered with those 34 reference sequences with 82% bootstrap support value in the phylogenetic tree (Figure 3), of which 29 sequences were from *C. vulgaris* organisms NJ-7, CCAP 211/19, CCAP 211/35, NIES-227, KNUA027, CCAP 211/75, CCAP 211/82, CCAP 211/74, CCAP 211/21B, CCAP 211/110, CCAP 211/11S, ACSSI 335, Ab5, ACSSI 249, ACSSI 374, ACSSI 378, S3, ChloN4, ZS1, ACSSI 361, CCAP 211/109, SAG 211-11b, CCAP 211/21A, CCAP 211/81, and CCAP 211/80, and five sequences were from *Neodesmus cf. pupukensis* CCAP 211-52, *Neochloris aquatica* CCAP 254/5, *Chlamydomonas chlamydogama* CCAP 11/48B, *Marvania coccoides* CCAP 251/1A, and *Chloroidium saccharophilum* CCAP 211/48 (dark gray background in Figure 3). In addition, two reference sequences with different accession numbers were from *C. vulgaris* organisms SAG 211-11b, CCAP 211/21A, CCAP 211/81, and CCAP 211/80, respectively (light gray background in Figure 3). Thus, the microalgal strain S3 was named *C. vulgaris* S3.

### Influence of microalgal fertilizers on soil properties

All soils treated with M9VL, M9VD, S3D, and S3L had higher SOM, TC, and C:N ratio than the control and urea soils (Table 1). The M9VL soil had the highest TC and C:N ratio and significantly increased TC (13.10 and 14.46%) and C:N ratio (6.96 and 11.73%) compared to the control and urea soils, respectively (*p* < 0.05) (Table 2). The M9VD soil had the highest SOM and the second highest C:N ratio and significantly increased SOM (29.15 and 18.22%) and C:N ratio (6.06 and 10.79%) compared to control and urea soils, respectively (*p* < 0.05) (Table 2). In addition, the M9VD soil had significantly higher TC (8.43%) than the urea soil (*p* < 0.05), and the M9VL soil had significantly higher OM (23.10%) than the control soil (Table 2). The S3D soil had significantly higher TC (11.31 and 12.65%) and C:N ratio (5.24 and 9.93%) than the control soil and the urea soil (*p* < 0.05), respectively, and the S3L soil had significantly higher TC (10.24%) and C:N ratio (7.58%) than in the urea soil (*p* < 0.05) (Table 2). Although the soils treated with microalgal fertilizer did not exhibit a significant increase in TN, M9VL, S3D, and S3L soils did exhibit an increase in TN by approximately 2.31–5.56% compared to the control soil and the urea soil (Table 1). Soil pH decreased in all the soils treated with the microalgal fertilizers M9VL, M9VD, S3D, and S3L compared to that in the control and urea soils, and the M9VL, M9VD, and S3D soils had significantly lower pH than the control soil (*p* < 0.05), and the M9VD soil also had significantly lower pH than the urea soil (*p* < 0.05) (Table 1).

**TABLE 1:** Soil properties in different fertilization treatments (Mean ± standard deviation).

Treatment	Soil properties				
	SOM (g kg^–1^)	TN (g kg^–1^)	TC (g kg^–1^)	C:N	pH
M9VL	25.42 ± 1.06ab	1.33 ± 0.05a	19.00 ± 1.82a	14.29 ± 1.18a	7.89 ± 0.04bc
M9VD	26.67 ± 1.77a	1.27 ± 0.06a	18.00 ± 0.17ab	14.17 ± 0.61a	7.88 ± 0.02c
S3L	22.96 ± 1.53bc	1.33 ± 0.08a	18.30 ± 0.70ab	13.76 ± 0.99ab	7.95 ± 0.03abc
S3D	23.71 ± 1.12abc	1.33 ± 0.06a	18.70 ± 1.25a	14.06 ± 0.37a	7.89 ± 0.02bc
Urea	22.56 ± 0.26bc	1.30 ± 0.01a	16.60 ± 0.35c	12.79 ± 0.27c	7.98 ± 0.02ab
Control	20.65 ± 0.79c	1.26 ± 0.06a	16.80 ± 0.29bc	13.36 ± 0.85bc	8.00 ± 0.036a
BP	22.28 ± 1.01bc	1.28 ± 0.07a	17.70 ± 0.35abc	13.83 ± 0.96ab	7.97 ± 0.04ab

M9VL, living *Chlamydomonas applanata* M9V; M9VD, dead *Chlamydomonas applanata* M9V; S3L, living *Chlorella vulgaris* S3; S3D, dead *Chlorella vulgaris* S3; Urea, with urea fertilizer; Control, without fertilizer; BP, before planting; SOM, soil organic matter; TC, total carbon; TN, total nitrogen. Values followed by different letters in the same column are significantly different based on Tukey’s test (*p* < 0.05).

**TABLE 2:** Influence of microalgal fertilizers on the wheat growth parameters and soil properties.

	Comparing to the control treatment				Comparing to the urea treatment			
	M9VL	M9VD	S3L	S3D	M9VL	M9VD	S3L	S3D
Shoot fresh weight	+ + + + 166.77%*	+ + 87.86%*	+ 16.93%	+ + + 88.21%*	+ + + + 125.68%*	+ + 58.92%*	−*CPSTABLEENTER*−1.08%	+ + + 59.73%*
Root dry weight	+ + + + 188.89%*	+ + + 122.22%*	+ 11.11%	+ + 111.11%	+ + + + 77.35%*	+ + + 36.43%*	− 31.79%	+ + 29.60%
Leaf length	+ + + + 26.88%*	+ + 13.48%	− 2.76%	+ + + 15.26%	+ + + + 14.56%	+ + 2.46%	− 12.20%	+ + + 4.07%
Root length	+ + + + 46.04%*	+ + 12.13%	+ 7.88%	+ + + 27.26%	+ + + + 43.93%*	+ + 10.52%	+ 6.33%	+ + + 25.43%
Chlorophyll *a*	+ + + + 257.81%*	+ + + 183.81%*	+ 16.78%	+ + 169.48%*	+ + + + 82.23%*	+ + + 44.55%*	− 40.52%	+ + 37.25%*
Chlorophyll *b*	+ + + + 269.00%*	+ + + 140.41%*	+ 26.86%	+ + 107.10%*	+ + + + 247.27%*	+ + + 126.25%*	+ 19.39%	+ + 94.90%*
OM	+ + + 23.10%*	+ + + + 29.15%*	+ 11.19%	+ + 14.82%	+ + + 12.68%	+ + + + 18.22%*	+ 1.77%	+ + 5.10%
TC	+ + + + 13.10%*	+ 7.14%	+ + 8.93%	+ + + 11.31%*	+ + + + 14.46%*	+ 8.43%*	+ + 10.24%*	+ + + 12.65%*
C:N	+ + + + 6.96%*	+ + + 6.06%*	+ 2.99%	+ + 5.24%*	+ + + + 11.73%*	+ + + 10.79%*	+ 7.58%*	+ + 9.93%*

M9VL, M9VD, S3L, and S3D indicate microalgal fertilizer treatments of living *Chlamydomonas applanata* M9V, dead *Chlamydomonas applanata* M9V, living *Chlorella vulgaris* S3, and dead *Chlorella vulgaris* S3, respectively. The “*” means a significant increase compared to the control or urea treatments. The “+” and “−,” respectively, mean positive and negative influence on the wheat growth parameters and soil properties, and the counts of “+” indicate the degree of positive effects.

### Influence of microalgal fertilizers on wheat growth parameters

**FIGURE 4:**
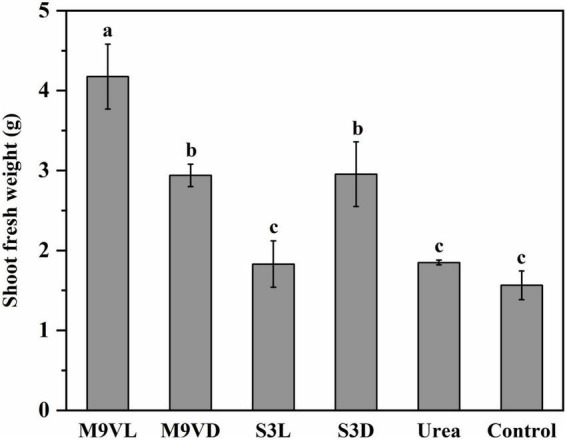
Effects of microalgal fertilizers and urea fertilizer on shoot fresh weight of wheat. The data are expressed as mean, and the error bars represent the standard deviation (SD). The significance level was defined as *p* < 0.05 using Tukey’s test. The M9VL, M9VD, S3L, S3D, urea, and control indicated microalgal fertilizer treatments living *Chlamydomonas applanata* M9V, dead *Chlamydomonas applanata* M9V, living *Chiarella vulgaris* S3, dead *Chlorella vulgaris* S3, urea fertilizer, and control without fertilizer, respectively.

**FIGURE 5:**
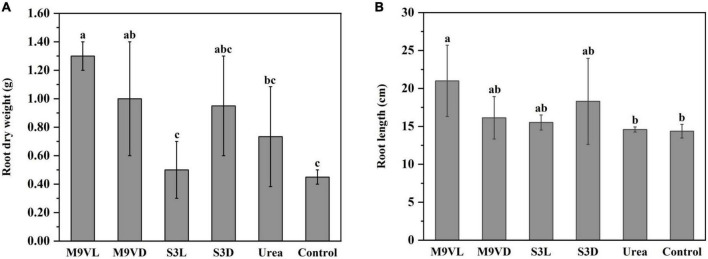
Effects of microalgal fertilizers and urea fertilizer on root dry weight **(A)** and root length **(B)** of wheat. The data are expressed as mean, and the error bars represent the standard deviation (SD). Significance was defined as *p* < 0.05 using Tukey’s test. The M9VL, M9VD, S3L, S3D, urea, and control indicated microalgal fertilizer treatments living *Chlamydomonas applanata* M9V, dead *Chlamydomonas applanata* M9V, living *Chlorella vulgaris* S3, dead *Chlorella vulgaris* S3, urea fertilizer, and control without fertilizer, respectively.

**FIGURE 6:**
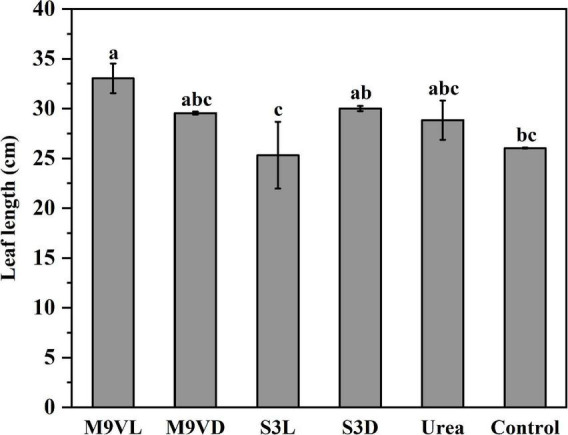
Effects of microalgal fertilizers and urea fertilizer on leaf length in wheat. The data are expressed as mean, and the error bars represent the standard deviation (SD). Significance was defined as *p* < 0.05 level using Tukey’s test. The M9VL, M9VD, S3L, S3D, urea, and control indicated microalgal fertilizer treatments living *Chlamydomonas applanata* M9V, dead *Chlamydomonas applanata* M9V, living *Chlorella vulgaris* S3, dead *Chlorella vulgaris* S3, urea fertilizer, and control without fertilizer, respectively.

Totally, M9VL was effective with respect to wheat growth promotion based on parameters, such as shoot fresh weight, root dry weight, leaf length, and root length, followed by M9VD and S3D, while the lowest fresh weight was observed in the control (Figures 4–6 and Table 2). M9VL significantly increased the shoot fresh weight (166.77 and 125.68%), root dry weight (188.89 and 77.35%), and root length (46.04 and 43.93%), respectively, compared to the control and urea treatments (*p* < 0.05), and also significantly increased the leaf length (26.88%) compared to the control (*p* < 0.05) (Table 2). M9VD significantly increased shoot fresh weight (87.86 and 58.92%) and root dry weight (122.22 and 36.43%) compared to the control and urea treatments (*p* < 0.05), and S3D significantly increased shoot fresh weight (88.21 and 59.73%), respectively, compared to the control and urea treatments (*p* < 0.05) (Table 2). However, S3L showed a slight increase in shoot fresh weight (16.93%), root dry weight (11.11%), and root length (7.88%) compared to the control, and a slight reduction in leaf length (2.76%) compared to the control (Table 2).

### Influence of microalgal fertilizers on chlorophyll *a/b* and carotenoid contents of wheat leaf

**FIGURE 7:**
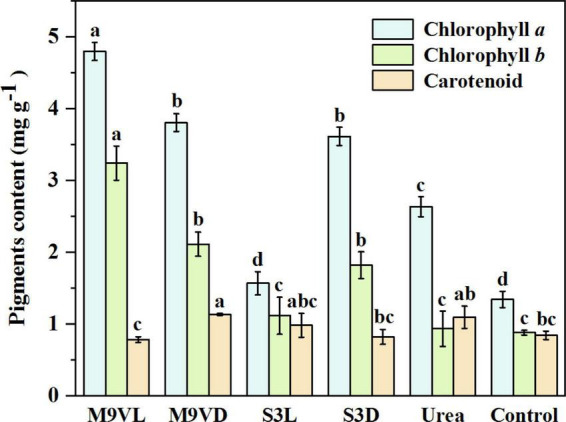
Effects of microalgal fertilizers and urea fertilizer on chlorophyll *alb*, and carotenoid contents in a wheat leaf. The data are expressed as mean, and the error bars represent the standard deviation (SD). Significance was defined as *p* < 0.05 using Tukey’s test. The M9VL, M9VD, S3L, S3D, urea, and control indicated microalgal fertilizer treatments living *Chlamydomonas applanata* M9V, dead *Chlamydomonas applanata* M9V, living *Chlorella vulgaris* S3, dead *Chlorella vulgaris* S3, urea fertilizer, and control without fertilizer, respectively.

The highest chlorophyll *a* and *b* contents of wheat leaves were recorded from M9VL, followed by M9VD and S3D, while the lowest contents were obtained in the control (Figure 7). Compared with the control, M9VL, M9VD, and S3D significantly increased the chlorophyll *a* (by 257.81, 183.81, and 169.48%, respectively) and *b* contents in wheat leaves (by 269.00, 140.41, and 107.10%, respectively) (Table 2). Compared with the urea, M9VL, M9VD, and S3D significantly increased the chlorophyll *a* content (by 82.23, 44.55, and 37.25%, respectively) and *b* contents in wheat leaves (by 247.24, 126.25, and 94.90%, respectively) (Table 2). In addition, the chlorophyll *a* and *b* contents of M9VL were significantly higher than those of the other treatments (*p* < 0.05), and these contents were significantly higher in M9VD and S3D than those in S3L, urea, and control (*p* < 0.05) (Figure 7). For the carotenoid content in wheat leaves, the highest content was obtained from M9VD, followed by urea and S3L, while the lowest was obtained from M9VL (Figure 7). A significantly higher carotenoid content was observed in M9VD than in the case of the control, S3D, and M9VL, and a significantly higher content was observed in the urea treatment than in the case of M9VL (*p* < 0.05) (Figure 7).

## Discussion

### Application of microalgae for crop growth promotion

According to the results, *C. applanata* M9V and *C. vulgaris* S3 isolated in the present study, both in their living and dead forms (M9VL, M9VD, S3L, and S3D), had different degrees of positive influence on wheat growth in the pot experiment (Figure 1 and Table 2). M9VL was effective with respect to wheat growth promotion, followed by M9VD and S3D as measured based on shoot fresh weight, root dry weight, leaf length, root length, and photosynthetic pigment (chlorophyll *a* and *b*) content (Figures 4–7 and Table 2). These growth-promoting characteristics resulted in thicker stalks, broader, longer, and darker green leaves, and a deeper developed root system in wheat (Figure 1 and Table 2), which might herald higher yield productivity at the mature stage of wheat. The use of microalgae as fertilizers in agricultural crop production is mainly focused on the cyanobacterial members especially *Nostoc* and *Anabaena* that are capable of fixing atmospheric nitrogen (Garcia-Gonzalez and Sommerfeld, 2016; Renuka et al., 2016). Green algal members of microalgae, such as *Acutodesmus dimorphus*, *C. vulgaris*, *Scenedesmus quadricauda*, *Chlamydomonas reinhardtii*, *Chlorella sorokiniana*, *Asterarcys quadricellulare, Dunaliella salina*, and *Chlorella ellipsoidea*, have been gradually investigated their fertilizer properties on plants, i.e., wheat, maize, tomato, potato, and lettuce (Faheed and Abd-El Fattah, 2008; Garcia-Gonzalez and Sommerfeld, 2016; El Arroussi et al., 2018; Barone et al., 2019; Martini et al., 2021; Mutale-Joan et al., 2021; Cordeiro et al., 2022). These green algal biomass or extracts could positively affect the growth of plants through growth phytohormones, exopolysaccharides, and nutrient availability (Rana et al., 2012; Renuka et al., 2016; El Arroussi et al., 2018).

In this work, we speculate that the growth-promoting effect of microalgae M9V and S3 on wheat might mainly be attributed to the increased soil nutrient contents and microalgae-derived bioactivity. Microalgal biomass is a carbon-rich residue comprising carbohydrates, lipids, proteins, and diverse molecules, which has benefits, such as improved soil health, stability of soil aggregates, soil water retention, carbon sequestration, and prevention of nutrient losses (Metting and Rayburn, 1983; Anand et al., 2015; Maurya et al., 2016; Behera et al., 2021). SOM is responsible for storing nutrients and maintaining soil structure, which plays an important role in soil quality and crop nutrient supply in the agro-ecosystem (Menšík et al., 2018). In the current study, microalgae M9V and S3 contained 39.32 and 35.52% carbon, respectively (Table 3). Using microalgae M9V and S3 in their living and dead forms as biofertilizers increased the amount of SOM, TC, and the C:N ratio compared to that in the individual urea and control treatments or a combination of the two (Tables 1, 2). The microalgae M9V and S3 in their dead forms had slightly higher SOM content than their living forms. Specifically, M9VD had higher SOM content (1.25 g kg^–1^) than M9VL, S3D had higher SOM content (0.75 g kg^–1^) than S3L. As for the nitrogen, not much significant increase in the soil TN content was observed in this study. The M9VL, S3D, and S3L treatments increased the TN compared to the control and urea treatments (0.07 g kg^–1^, 5.56%; 0.03 g kg^–1^, 2.31%, respectively). Furthermore, M9VD treatment increased the TN (0.01 g kg^–1^, 0.79%) compared to the control treatment, suggesting that the continuous application of microalgae may have significant long term benefits with respect to improving the soil TN (Table 1). With respect to the vast majority of nitrogen-fixing organisms [prokaryotic bacteria or cyanobacteria (blue-green algae)] (Garcia-Gonzalez and Sommerfeld, 2016; Atnoorkar, 2021), we speculated that the increased nitrogen came from the chemical elements of microalgal cells or the recycling of elements directly or indirectly affected by microalgae. Finally, certain microalgal extracts that enhance the growth of crops have been found to contain high levels of macro- and micronutrients (Tarakhovskaya et al., 2007; Renuka et al., 2016). In this study, other nutrient elements including P, K, Fe, Zn, and Mn, released from the biomass of microalgae M9V and S3, in the background of autoclaving at 121°C, might also play an essential role in wheat growth promotion (Table 3).

**TABLE 3:** Elemental composition of microalgal biomass of *Chlamydomonas applanata* M9V and *Chlorella vulgaris* S3.

Elemental composition	*Chlamydomonas applanata* M9V	*Chlorella vulgaris* S3
N (%)	5.49	4.80
C (%)	39.32	35.52
P (%)	2.06	1.68
K (%)	0.76	0.57
Fe (mg kg^–1^)	811	966
Zn (mg kg^–1^)	68	53
Mn (mg kg^–1^)	435	285

Studies have shown that microalgae can promote crop growth by producing plant hormones (auxins, gibberellins, and cytokinins), amino acids, vitamins, and antifungal and antibacterial compounds (Maurya et al., 2016; Ronga et al., 2019; Behera et al., 2021). Considering that M9VL resulted in much higher values for shoot fresh weight, root dry weight, leaf length, root length, and photosynthetic pigment (chlorophyll *a* and *b*) content than M9VD, and did not result in any significant difference in soil nutrients compared to M9VD (Figures 4–7 and Table 2), we found that the living *C. applanata* M9V tended to possess some bioactivity with respect to positively affecting wheat growth. Consequently, the positive influence of *C. applanata* M9V on crop growth might mainly be attributed to excellent biological activity followed by the improvement in the soil nutrient contents. On the contrary, the positive influence of *C. vulgaris* S3 on crops might mainly be attributed to the improvement of the soil nutrient contents because S3D resulted in much better results for most wheat growth parameters than S3L (Figures 4–7 and Table 2). Although S3L did not result in any obvious growth promotion, it had an effect similar to that of urea and control. Reports indicate that plant growth is negatively influenced by much higher concentrations of microalgal extracts, with lower seed germination, fewer lateral roots, and shorter shoot length (Kumar and Sahoo, 2011; Hernández-Herrera et al., 2014).

### Application of microalgae as urea alternatives

Nitrogen is essential for crop growth and significantly affects root and leaf growth and yield by affecting photosynthesis and the synthesis of proteins and nucleic acids during crop production. However, the low utilization rate of 30–50% of the nitrogen in the soil environment and emission of greenhouse gases (nitrous oxides), and leaching of nitrogen into the groundwater caused by excess usage of chemical fertilizers is very prominent (Fan et al., 2004; Quaggio et al., 2005; Chien et al., 2009; Shcherbak et al., 2014; Maurya et al., 2016). Urea is the most commonly used nitrogenous chemical fertilizer in crop production. The results of this study showed that the wheat plants treated with the microalgal biomass showed better results in terms of shoot fresh weight, root dry weight, leaf length, root length, and photosynthetic pigment (chlorophyll *a* and *b*) content than the wheat plants treated with urea fertilizer and no fertilizer (Figures 4–7 and Table 2). Thus, it could be estimated that the microalgal biomass of M9V and S3 could be used as a substitute for a certain proportion of chemical urea fertilizer, which would help reduce the use of chemical fertilizers. With respect to the conventional fertilizer application rate of approximately 400–700 kg N ha^–1^ in the winter-wheat/summer-maize rotation system in the North China Plain (Zhao et al., 2009), microalgae might achieve approximately 17–30% substitution of chemical fertilizer application, which would have the potential to maintain crop yields, and alleviate a series of environmental pollution problems.

Additionally, all the microalgal fertilizer treatments, including M9VL, M9VD, S3L, and S3D, increased the root length of wheat compared to the urea and control treatments (Figures 1, 5B). In addition, all the microalgal fertilizer treatments except the S3L treatment increased the root dry weight of wheat compared to the urea and control treatments, while the S3L treatment resulted in a higher root dry weight than the control treatment but not the urea treatment (Figure 5A). Furthermore, M9VL treatment significantly promoted the growth of wheat roots, including root dry weight and length compared to the urea and control treatments, and M9VD treatment significantly promoted root dry weight compared to the control treatment (Figures 1, 5). The promotion of root growth using microalgal biomass would accelerate the uptake of nutrients from the soil, thereby increasing nitrogen utilization and alleviating the leaching of nitrogen into the groundwater, a phenomenon that is conducive to coordinated and sustainable environmental protection and agricultural production (Martini et al., 2021; Mutale-Joan et al., 2021).

### Prospects of applying microalgae for crop production in a nature-friendly way

Excessive use of chemical fertilizers results in decreased crop yields and significant soil pollution (Rahman and Zhang, 2018). Thus, innovative technologies that would increase agricultural yields while minimizing inputs and environmental pollution are a crucial concern (Tilman et al., 2002; Foley et al., 2011; Garcia-Gonzalez and Sommerfeld, 2016; Singh et al., 2016). Biofertilizers are products that contain living microorganisms, natural compounds, or substances derived from organisms, such as bacteria, fungi, and algae, which can improve soil chemical and biological properties, stimulate plant growth, and restore soil fertility (Abdel-Raouf et al., 2012; Ronga et al., 2019). A few reports provide insights into the potential use of microalgae as biofertilizers, considering that microalgae are rich in biochemical components, bioactive metabolites, micronutrients, and macronutrients, such as proteins, carbohydrates and lipids, phytohormones, carotenoids, and vitamins, which would benefit plant growth with greater nutrient uptake, higher biomass accumulation, and greater crop yields (Shaaban, 2001; Faheed and Abd-El Fattah, 2008; Maurya et al., 2016; Behera et al., 2021). Microalgal biofertilizers provide a possible alternative to chemical fertilizers as they are considered environmentally friendly and economically feasible (Kawalekar, 2013; Garcia-Gonzalez and Sommerfeld, 2016). Not only do they increase agricultural productivity, but they have also been shown to decrease environmental pollution (Kawalekar, 2013; Garcia-Gonzalez and Sommerfeld, 2016). Meanwhile, the use of microalgal biofertilizers as a substitute for chemical fertilizers results in improved soil health, soil aggregate stabilization, enhanced soil water retention, nutrient loss prevention, and carbon sequestration (Metting and Rayburn, 1983; Anand et al., 2015; Maurya et al., 2016).

In recent years, although increasing worldwide interest in the use of microalgae in ecological crops production, poor soils remediation and adverse conditions of a changing climate has been observed, microalgal resources with biofertilizer properties remain largely unexploited (Grzesik and Romanowska-Duda, 2014; Ronga et al., 2019). In this study, microalgal strains *C. applanata* M9V and *C. vulgaris* S3 promote crop growth and increase soil nutrient contents, favoring the application of microalgae as biofertilizers to reduce the usage of chemical fertilizers, thereby promoting sustainable crop production. Previous studies have shown that *C. vulgaris* seems to be a relatively common strain, the same specie as S3 in this study, which can enhance growth parameters and strengthen the metabolic aspects (Shaaban, 2001; Faheed and Abd-El Fattah, 2008; Feng et al., 2022). To the best of our knowledge, the *C. applanata* M9V might be the first *applanata* species of the *Chlamydomonas* genus that can promote crop growth. At present, the *reinhardtii* species of the *Chlamydomonas* genus are most commonly used as model organisms for basic research and biotechnological applications (Yang et al., 2019), and a recent study showed that *C. reinhardtii* strain 4a+ from the Culture Collection of Algae at Goettingen University (Germany) promotes the development of the maize root system (Martini et al., 2021). The *C. applanata* M9V exploited in this study has enriched the existing microalgal resources, and it might have huge development potential and broad application prospects for generating high-quality agriculture.

## Conclusion

In the present study, *C. applanata* M9V and *C. vulgaris* S3, both in their living and dead forms (M9VL, M9VD, S3L, and S3D), were used as alternatives to chemical fertilizers for wheat growth. The results suggested that M9VL, M9VD, and S3D as microalgal fertilizers performed as well as and even better than a certain amount of chemical urea fertilizer with respect to wheat growth promotion, while S3L treatment exhibited an effect similar to that of the urea treatment and a slightly better effect than the control treatment, except in the case of leaf length. Moreover, all the microalgal fertilizer treatments improved soil properties, such as SOM, TC, and C:N ratio (compared to chemical urea fertilizer and the control without fertilizer). In particular, M9VL performed the best with respect to wheat growth promotion in the context of parameters, such as plant fresh weight, root dry weight, leaf length, root length, and plant photosynthetic pigment (chlorophyll *a* and *b*) content, which might mainly be attributed to its excellent biological activity and improved soil nutrient contents. The use of microalgae for crop growth promotion and as urea alternatives is a feasible strategy in the context of crop production. The *C. applanata* M9V obtained in this study has the potential for further development as a substitute for partial chemical fertilizers with a positive and eco-friendly influence on wheat growth and soil nutrient contents.

## Data availability statement

The datasets presented in this study can be found in online repositories. The names of the repository/repositories and accession number(s) can be found at: https://www.ncbi.nlm.nih.gov, MK793578 and MK652782.

## Author contributions

MYS designed and performed all experiments, analyzed the data, and wrote the first draft of the manuscript. YPT and XGW contributed to the project administration of the study and reviewed and edited the manuscript. XZW performed the pot experiments, analyzed the data, and revised the manuscript. All authors read and approved the submitted version.

## Footnotes

1
http://www.ncbi.nlm.nih.gov/BLAST
